# A Potential Role of YAP/TAZ in the Interplay Between Metastasis and Metabolic Alterations

**DOI:** 10.3389/fonc.2020.00928

**Published:** 2020-06-11

**Authors:** Hirohito Yamaguchi, Ghina M. Taouk

**Affiliations:** Cancer Research Center, College of Health and Life Sciences, Qatar Biomedical Research Institute (QBRI), Hamad Bin Khalifa University (HBKU), Qatar Foundation (QF), Doha, Qatar

**Keywords:** YAP, TAZ, cancer, metabolism, metastasis

## Abstract

Yes-Associated Protein (YAP) and Transcriptional Co-activator with PDZ-binding Motif (TAZ) are the downstream effectors of the Hippo signaling pathway that play a crucial role in various aspects of cancer progression including metastasis. Metastasis is the multistep process of disseminating cancer cells in a body and responsible for the majority of cancer-related death. Emerging evidence has shown that cancer cells reprogram their metabolism to gain proliferation, invasion, migration, and anti-apoptotic abilities and adapt to various environment during metastasis. Moreover, it has increasingly been recognized that YAP/TAZ regulates cellular metabolism that is associated with the phenotypic changes, and recent studies suggest that the YAP/TAZ-mediated metabolic alterations contribute to metastasis. In this review, we will introduce the latest knowledge of YAP/TAZ regulation and function in cancer metastasis and metabolism, and discuss possible links between the YAP/TAZ-mediated metabolic reprogramming and metastasis.

## Introduction

Metastasis is the spread of cancer cells from primary tumors to distinct organs and a major cause of cancer-related death. Therefore, targeting metastasis is expected to be an effective therapeutic strategy to improve the survival of cancer patients. Metastasis is a complex process including various distinct steps, and cancer cells need to gain diverse abilities in each step that are regulated by a variety of cellular signaling pathways (1). Thus, the understanding of regulatory mechanisms of metastasis is particularly crucial for targeting it.

During tumor development, cancer cells are exposed to dynamic changes of tumor microenvironment such as availability of nutrient and oxygen. Hence, cancer cells need to adapt their metabolism to these changes to develop tumors (2). Emerging evidence has suggested that metabolic reprogramming also plays a significant role in metastasis, during which cancer cells are exposed to different environment from primary tumors to distant metastatic sites (3).

Yes-Associated Protein (YAP) and Transcriptional Co-activator with PDZ-binding Motif (TAZ) are the transcriptional co-regulators and downstream effectors of the Hippo signaling pathway (4). Numerous studies using mouse models have demonstrated that YAP/TAZ promotes tumor development, progression, and metastasis (4). At cellular level, YAP/TAZ regulates cell cycles, migration, invasion, anchorage-independent growth, epithelial-mesenchymal transition (EMT), and stemness. Because all these functions of YAP/TAZ are crucial for various steps of metastasis, YAP/TAZ serves as a central regulator of metastasis. Besides, it has gradually been recognized that YAP/TAZ regulates cellular metabolic reprograming to adapt the various tumor microenvironment (5).

Based on the fact that YAP/TAZ has the ability to regulate both metastasis and metabolism, it is expected that YAP/TAZ functions as a central hub for metastasis by regulating both metabolic adaptation and phenotypic changes required for metastasis. Thus, in this review, we will introduce the latest knowledge of YAP/TAZ regulation and function in cancer metastasis and metabolism, and discuss possible links between the YAP/TAZ-mediated metabolic reprogramming and metastasis.

## Regulation OF YAP/TAZ

**Figure 1:**
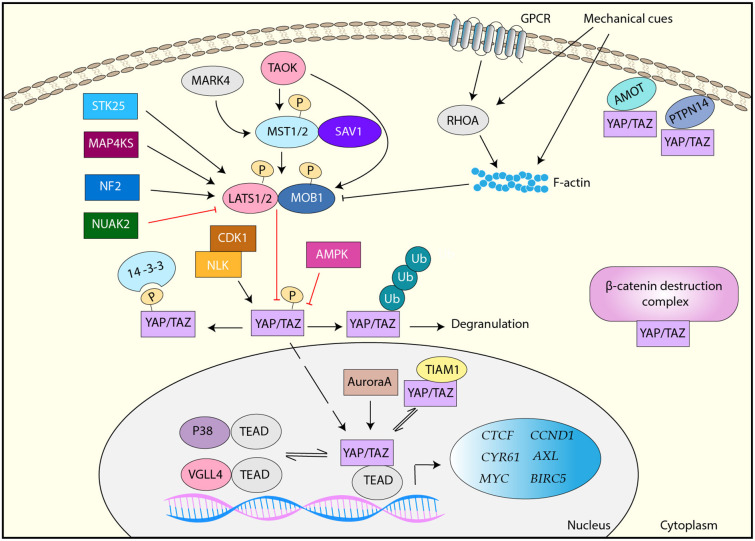
Regulation of YAP/TAZ. YAP/TAZ is primarily regulated by the canonical Hippo pathway, MST1/2-SAV1 > LATS1/2-MOB1. LATS1/2 phosphorylates YAP/TAZ and inactivate it via either 14-3-3-mediated cytoplasmic sequestration or ubiquitination and proteasome-mediated degradation. Unphosphorylated YAP/TAZ translocates to the nucleus, where it interacts with TEAD transcription factors and induces target genes. LATS1/2 is activated by TAOK, STK25, MAP4KS, and NF2, but inactivated by mechanical cues- and GPCR-RHOA-mediated F-actin and NUAK2. MST1/2 is activated by MARK4 and TAOK. YAP/TAZ is also regulated by LATS-independently. AMOT and PTPN14 interact with YAP/TAZ and sequester it in the plasma membrane. YAP/TAZ is inhibited by the β-catenin destruction complex or TIAM1 via a direct interaction. YAP/TAZ is also directly phosphorylated and regulated by AMPK, CDK1, NLK, Aurora A, and several RTKs. In addition, VGLL4 and p38 interact TEAD and inhibit YAP/TAZ activity.

YAP/TAZ is the downstream effector of the Hippo signaling pathway, which is an evolutionally conserved kinase cascade (Figure 1) (6, 7). The key regulators of the Hippo pathway include two kinases mammalian sterile 20-like kinase 1 and 2 (MST1/2) and large tumor suppressor kinase 1 and 2 (LATS1/2) and their regulatory subunits SAV1 and MOB1, respectively (Figure 1) (6). MST1/2 forms a complex with SAV1, which phosphorylates and activates the downstream LATS1/2-MOB1 complex (6). Activated LATS1/2 phosphorylates YAP/TAZ at multiple serine/threonine residues. Upon phosphorylation, YAP/TAZ interacts with 14-3-3, resulting in its cytoplasmic sequestration (8). Alternatively, phosphorylated YAP/TAZ is ubiquitinated by E3 ligase β-TrCP and undergoes proteasome-dependent degradation (9, 10). Thus, MST1/2 and LATS1/2 are the negative regulators of YAP/TAZ. Unphosphorylated YAP/TAZ moves to the nucleus, where YAP/TAZ interacts with (TEA domain) TEAD family transcription factors and functions as a transcription co-activator (11, 12). Major YAP/TAZ target genes include molecules that are related to cell growth, proliferation, and migration such as *CTGF, CYR61, MYC, AXL, BIRC5*, and *CCND1* (13, 14).

Various extracellular stimuli regulate YAP/TAZ through the Hippo signaling pathway-dependent and independent mechanisms. In particular, YAP/TAZ plays a central role in cellular mechanotransduction, which is a molecular process to convert various extracellular mechanical forces to cellular responses (15). Namely, mechanical cues from surrounding extracellular matrix (ECM) and cells are converted to biochemical signals to control gene expression and protein functions, resulting in cell proliferation, survival, differentiation, and migration (16). YAP/TAZ nuclear localization and activity are regulated by various mechanical cues, and activated YAP/TAZ is required for biological function induced by mechanical cues (17–20). Increased polymerized actin (F-actin) induced by mechanical cues contributes to YAP/TAZ activation through Hippo-dependent and independent mechanism, although the detail mechanism is still uncertain (21). Moreover, various G-protein-coupled receptors (GPCR) induces F-actin through RHOA activation, resulting in LATS1/2 inactivation and subsequent YAP/TAZ activation (22).

Besides, several upstream regulators of MST/LATS have been identified. Tao kinase (TAOK) phosphorylates and activates MST1 and LATS1/2 (23), and several mitogen-activated protein kinase kinase kinase kinase (MAP4K) family members directly phosphorylate and activate LATS1/2 (24, 25). A recent study has also shown that STK25 phosphorylates LATS1/2 and contribute to the activation of LATS1/2 in response to contact inhibition and cell detachment (26). In addition, tumor suppressor Neurofibromin 2 (NF2) is a critical activator of LATS1/2 (27). Therefore, these molecules ultimately inactivate YAP/TAZ. In contrast, NUAK2 phosphorylates LATS1 and inhibits it, thereby activating YAP/TAZ (28), while MAP/microtubule affinity-regulating kinase 4 (MARK4) can phosphorylate MST2 and SAV1, resulting in the disruption of MST2/SAV1/LATS1 complex and subsequent YAP/TAZ activation (29).

In addition to LATS-mediated phosphorylation, YAP/TAZ is regulated by a variety of molecules. Angiomotin (AMOT) and protein tyrosine phosphatase non-receptor type 14 (PTPN14) directly bind to YAP to sequester YAP in the plasma membrane (30, 31). Besides, it has been shown that YAP/TAZ is found in the β-catenin destruction complex and it is released from the complex upon Wnt ligand and translocates to the nucleus (32). Moreover, multiple kinases directly phosphorylate YAP/TAZ at the various sites that are different from LATS-mediated phosphorylation sites, and activate or inactivate it. For example, in response to cellular energy stress, AMP-activated protein kinase (AMPK) phosphorylates YAP and inactivates it (33, 34). CDK1 phosphorylates YAP during the G2–M phase of the cell cycle, and the CDK1-mediated YAP phosphorylation has been implicated to be linked to mitotic defects (35). In response to osmotic stress, NLK phosphorylates YAP and inhibits the interaction between YAP and 14-3-3, thereby enhancing its nuclear localization (36). Aurora A also interacts with and phosphorylates YAP in the nucleus, and contributes to YAP transcriptional activity (37). Furthermore, PYK2 is implicated to phosphorylate TAZ at its tyrosine residues and regulate its protein stability (38). Several receptor tyrosine kinases (RTKs) including FGFR, RET, and MERTK are also shown to phosphorylate YAP/TAZ and activate it (39).

A major function of YAP/TAZ is transcriptional regulation through DNA binding protein TEAD family proteins (40, 41). Several regulatory mechanisms for the YAP/TAZ-TEAD complex have been identified. VGLL4 inhibits YAP by competing with YAP for its TEAD-binding (42). TIAM1 interacts with YAP/TAZ and impairs the YAP/TAZ-TEAD interaction (43). Moreover, osmotic stress induces the interaction between TEAD and p38 MAP kinase, resulting in TEAD translocation to the cytoplasm and YAP/TAZ inactivation (44). In addition to these factors, YAP/TAZ has been reported to interact with other transcription regulators such as RUNX2 and AP-1, cooperate to regulate the expression of target genes (45). Therefore, YAP/TAZ is regulated by a variety of stress and stimuli through multiple mechanisms (Figure 1).

## The Role of YAP/TAZ in Cancer Progression

YAP/TAZ functions as an oncogenic transcription factor in majority of solid tumors. Numerous studies have demonstrated that high expression and/or nuclear localization of YAP/TAZ in cancer cells are correlated with poor clinical outcomes in many cancer types such as lung, colorectal, breast, liver, gastric, pancreatic, prostate, endometrial, esophageal, bladder, and ovarian cancer (4, 46, 47). Moreover, it has been shown that the signatures of YAP/TAZ regulated gene expression are correlated with poor prognosis of lung and breast cancers (48, 49). Moreover, gene inactivation by mutations or deletions in multiple upstream regulators of the Hippo pathway, such as *LATS1/2, PTPN14*, and *NF2* has been identified in several cancers including mesothelioma, skin basal cell carcinoma, and bladder and colon cancer (50–52).

In addition to retrospective analysis of clinical samples, numerous animal studies have also verified the function of YAP/TAZ as a bona-fide oncogenic factor. For example, conditional knockout of MST1/2 in liver or liver-specific YAP overexpression induces spontaneous liver cancer (53–55). Ectopic expression of YAP promotes tumor formation and progression in the genetically engineered mouse (GEM) model of lung cancer (56). Moreover, genetic inactivation of YAP reduces or suppresses tumor formation in various GEM models of lung, breast, colon, and pancreatic cancer (56–59).

At the cellular level, YAP/TAZ is involved in diverse functions required for tumor progression. For example, YAP/TAZ promotes cell proliferation by upregulating many genes involved in cell cycle control such as *CCND1, CDK1, CDC25*, and *MCMs* (40, 60, 61). YAP/TAZ also promotes anchorage-independent growth in various cancer cells (37, 62, 63).

To establish tumors, cancer cells need to escape from immune surveillance. Recently, it has been demonstrated that YAP/TAZ is involved in immune evasion as well as the resistance to immune checkpoint inhibitors (64). For instance, YAP/TAZ has been shown to upregulate chemokine CXCL5 and CCL2, resulting in the recruitment of immune suppressive myeloid-derived suppressor cells (MDSC) and M2 macrophages, respectively (65, 66). YAP/TAZ also upregulates PD-L1 in cancer cells, which is a critical regulator for the immune checkpoints (67, 68).

Furthermore, some studies have indicated that YAP/TAZ is essential for the maintenance of cancer stem cells. For example, TAZ is required for maintenance of breast cancer stem cells (49), while in osteosarcoma, YAP functions downstream of SOX2 to maintain cancer stem cells (69). In basal like breast cancer cells, serum response factor (SRF) interacts with YAP and induces IL-6 expression, which promotes stemness of basal like breast cancer cells (70). In esophageal cancer cells, YAP induces SOX9, which is engaged in cancer stem cell properties (71, 72).

During cancer development, cancer cells require high energy and a large amount of building blocks to maintain their growth and proliferation. However, because of limited oxygen and nutrients in tumor microenvironment, cancer cells are exposed to various metabolic stress and need to adapt their metabolism to it (73). Recent studies have shown that YAP/TAZ is not only regulated by diverse metabolic signals, but also serves as a key regulator of various metabolic pathways (5).

**Figure 2:**
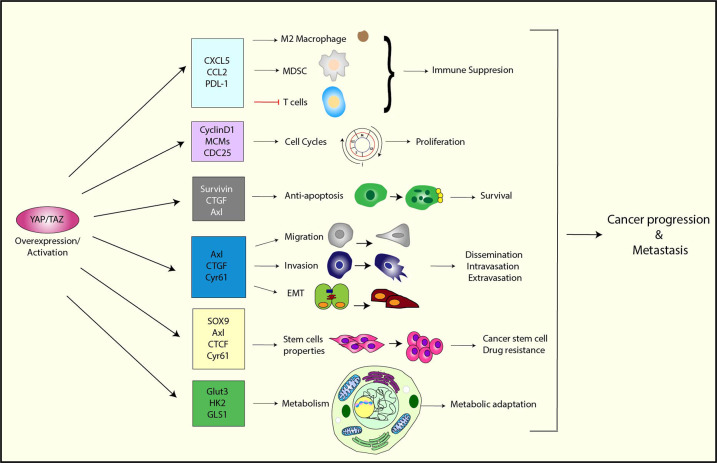
The function of YAP/TAZ in cancer progression and metastasis. YAP/TAZ promotes tumor progression and metastasis by upregulating a variety of its target genes that are involved in immune suppression, cell cycle control, anti-apoptosis, migration/invasion, EMT, stem cell properties, and metabolism.

Therefore, YAP/TAZ obviously plays an essential role in cancer progression (Figure 2). In addition to the functions that were discussed earlier, YAP/TAZ regulates various cellular functions that are essential for metastasis, such as cell migration, invasion, survival, and EMT. We will discuss these functions in detail in the next section.

## Metastasis Regulation by YAP/TAZ

It has been recognized that metastasis occurs through multiple steps known as the metastatic cascade. First, cancer cells gain the ability of migration and invasion, and these cells are dissociated from primary tumors to enter blood and lymphatic vessels. Then, the cells circulates in the blood stream, and some surviving cells extravasates at distant tissues and organs to form secondary tumors (1, 74).

Multiple retrospective analyses of clinical samples have shown that the upregulation or nuclear localization of YAP/TAZ is correlated with metastasis in a variety of cancer such as breast, colorectal, gastric, lung, liver, and pancreatic cancer (75–80). Similarly, the downregulation of upstream negative regulators of the Hippo pathway is also associated with metastasis in the various cancer (81–84).

Moreover, a number of *in vivo* studies have demonstrated the role of YAP/TAZ in metastasis. Overexpression or activation of YAP/TAZ promotes the ability of cancer cells to metastasize in xenograft mouse models of several cancer types (85–87). In contrast, inactivation of YAP/TAZ suppresses metastasis in several mouse models (87, 88).

At the cellular level, YAP/TAZ has been demonstrated to regulate multiple essential steps of the metastatic cascade (Figure 2). Cell migration and invasion abilities are crucial for cancer cell metastasis, and many studies have shown that overexpression and activation of YAP/TAZ promotes cell migration and invasion (85, 89–92), while the inhibition of YAP/TAZ reduces the migration and invasion abilities of cancer cells (91–93). Mechanistically, YAP/TAZ has been shown to control cell motility by limiting cytoskeletal and focal adhesion maturation (94). Moreover, YAP/TAZ promotes cell migration and invasion through the upregulation of various downstream targets, such as Axl, Cyr61, and receptor for hyaluronan-mediated motility (RHAMM) (89, 95, 96).

EMT is the process that epithelial cells lose their characteristics of cell polarity and cell-cell adhesion and gain mesenchymal cell properties, and associated with cell motility (97, 98). Because the initial step of metastasis is the detachment of cells from a tumor mass, EMT is considered to be the first event for metastasis (97, 99). Numerous studies have shown that YAP/TAZ is a key regulator of EMT. Overexpression of YAP/TAZ induce EMT (11, 12, 93, 100, 101), while the inhibition of YAP/ TAZ reverses EMT (93, 102, 103). Mechanistically, several YAP/TAZ targets such as Axl, Cyr61, and CTGF have been shown to have the ability to induce EMT and stemness (104–106). Moreover, YAP has been shown to interact with Smad2/3/4 and regulate the mRNA expression of EMT-inducing transcription factors, *Snail, Twist1*, and *Slug* (107). EMT-inducing transcription factor ZEB1 also interacts with YAP and enhances its transcription activity (108).

During metastasis, cancer cells get into blood vessels and circulate in a body. For cancer cells to metastasize, they need to evolve a mechanism, which helps them to survive during their circulation in blood vessels. Anoikis is the type of apoptotic cell death that is induced by cell detachment, and gaining an ability of resistance toward anoikis is critical for cancer cells to metastasize (109). It has been shown that YAP/TAZ is able to inhibit anoikis (84). Interestingly, platelets interacts with cancer cells, in which YAP is activated through RHOA-MYPT1-PP1-mediated YAP dephosphorylation (110). The platelets-mediated YAP activation contributes to resistance to anoikis and metastasis (110). Moreover, it has been shown that fluid shared stress activates YAP/TAZ, suggesting that YAP/TAZ is activated during circulation and the activated YAP/TAZ may contribute to cancer cell survival during metastasis (111, 112).

## The Role of YAP/TAZ in Glucose Metabolism

Glucose is an essential source of energy and building blocks for cells. It is metabolized through glycolysis, TCA cycle, and oxidative phosphorylation (OXPHOS) to produce ATP. In the presence of oxygen, normal cells primarily generate ATP through oxidative phosphorylation and exhibit reduced glucose consumption. However, cancer cells uptake high levels of glucose and convert it to lactate via glycolysis regardless of the presence of oxygen (aerobic glycolysis). The enhanced glycolysis, pentose phosphate, and hexosamine biosynthetic pathways support the energy and building blocks required for high replication of cancer cells (113).

**Figure 3:**
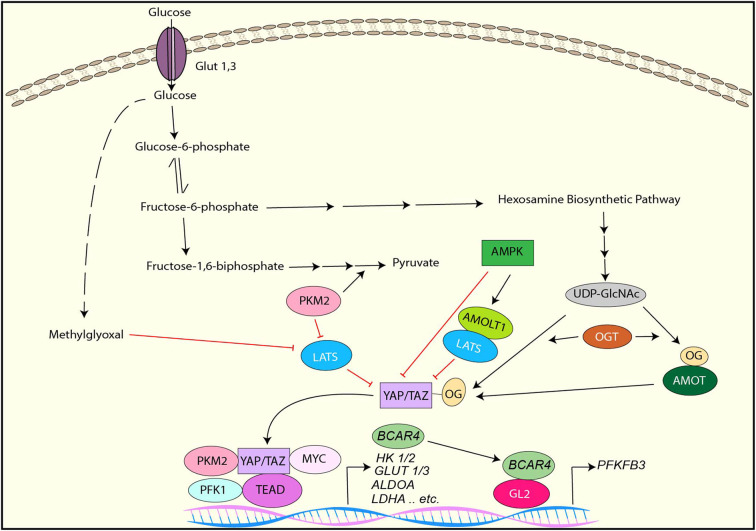
YAP/TAZ regulation by glucose metabolism and YAP/TAZ-targets for glucose metabolism. YAP/TAZ is inhibited by AMPK directly or indirectly through AMOTL1-mediated LATS activation. LATS is inhibited by PKM2 or Methylglyoxal, resulting in YAP/TAZ activation. YAP activation is also induced by direct O-GlcNAcylation or indirect O-GlcNAcylation of AMOT. In the nucleus, PKM2, PFK1, or Myc interacts with the YAP/TAZ-TEAD complex and enhances its transcriptional activity toward to the genes involved in glucose metabolism. YAP/TAZ induces lncRNA *BCAR4*, which interacts with GLI2 and induces *PFKFB3* transcription.

It has been demonstrated that YAP/TAZ is inactivated under the low glucose condition or by the inhibition of glycolysis while it is activated in response to high levels of glucose (33, 34, 114, 115). Several distinct mechanisms of glucose-mediated YAP/TAZ activation have been identified (Figure 3).

AMPK is a key cellular metabolic sensor that is activated by a high AMP to ATP ratio (116). As described earlier section, studies have shown that AMPK directly phosphorylates YAP and inhibits its transcriptional activity (33, 34). Also, these studies have indicated that LATS is also activated by glucose deprivation and contributes to YAP inactivation independently of AMPK, although the underlying mechanism is uncertain (33, 34). Another study has revealed that AMPK phosphorylates angiomotin-like 1 (AMOTL1), resulting in the stabilization of AMOTL1, which contributes to LATS-mediated YAP phosphorylation and inactivation (114).

O-GlcNAcylation is a posttranslational modification of proteins that is catalyzed by O-GlcNAc transferase (OGT) (117, 118). Uridine diphosphate N-acetylglucosamine (UDP-GlcNAc) is a donor sugar of O-GlcNAcylation and transferred to serine/threonine residues of myriad target proteins by OGT (117, 118). UDP-GlcNAc is synthesized from glucose through the hexosamine biosynthetic pathway, which links to cellular O-GlcNAcylation levels (118). Moreover, it has been shown that aberrant protein O-GlcNAcylation is associated with metabolic reprogramming in cancer (119). It has been reported that YAP is also O-GlcNAcylated in response to high levels of glucose (120, 121). One study has shown that YAP is O-GlcNAcylated at Serine 109 (S109) by OGT under high glucose, and S109-O-GlcNAcylation of YAP prevents YAP-LATS interaction, thereby activating YAP (121). In contrast, another study has revealed that O-GlcNAcylation of YAP at threonine 214 (T214), which is also induced by high levels of glucose, enhances its stability by inhibiting the β-TrCP-YAP interaction. Besides, T214 O-GlcNAcylation of YAP is associated with YAP tumorigenic activity (120). Moreover, it has been reported that AMOT is also O-GlcNAcylated, and AMOT expression and O-GlcNAcylation is enhanced by high levels of glucose (122). Interestingly, AMOT enhances YAP nuclear localization and transcriptional activity under the high glucose condition while AMOT inhibits them in normal levels of glucose, suggesting that AMOT O-GlcNAcylation is another mechanism of YAP/TAZ regulation by high level of glucose (122).

In addition to the above mentioned mechanism, phosphofructokinase (PFK1), which is one of the enzymes regulating glycolysis, has been implicated to regulate YAP/TAZ activity (115). Inhibition of glycolysis with 2-deoxy-glucose (2DG) reduces TEAD-YAP complex formation, thereby reducing YAP-mediated transcription. Activated PFK1 interacts with TEAD and form a complex with TEAD and YAP, resulting in stabilization of the YAP-TEAD complex and YAP activation (115).

Methylglyoxal (MG) is a byproduct of glycolysis that induces protein glycation and formation of advanced glycation end products (AGEs). A study has revealed that high MG-adducts and nuclear YAP are correlated in breast cancer, and MG induces YAP activation in breast cancer cells (123). Mechanistically, MG induces HSP90 glycation, which impairs its chaperon activity on LATS1, resulting in destabilization of LATS1 protein and subsequent YAP activation (123).

Pyruvate kinase M2 (PKM2) is a splice isoform of pyruvate kinase that plays an critical role in aerobic glycolysis in cancer (124). It has been reported that tyrosine 105-phosphorylated PKM2, which is induced by several tyrosine kinases, reduces LATS1 protein stability and downregulates it, thereby activating YAP (125). Furthermore, another report has shown that PKM2 directly interacts with YAP and enhances YAP transcriptional activity (126).

Because YAP/TAZ promotes cell growth and proliferation that require high nutrients, it is rational that YAP/TAZ is activated by high cellular glucose and inhibited under a limited energy source. However, in this context, it is also reasonable that YAP/TAZ controls glycolysis to support cell growth and proliferation. Indeed, emerging evidence has shown that YAP/TAZ regulates glycolysis by upregulating the key enzymes for the pathway as well as glucose transporters (Figure 3).

It has been demonstrated that knockdown of YAP/TAZ downregulates various genes involved in glycolysis including *GLUT3, HK1,HK2, PFKFB4, PFKP, GAPDH, PGK1, PGAM1, LDHA, PDHA1, and PDHB*, resulting in the downregulation of glycolysis and upregulation of mitochondrial oxidative phosphorylation in renal cell carcinoma cells (127). In cancer cells from a *KRAS* mutant pancreatic cancer GEM, deletion of YAP downregulates the expression of *HK2, ALDOA, GPADH, PGK1, PGAM1*, and *LDHA* genes (128). Interestingly, YAP cooperates with other transcription regulators to control many genes related to glycolysis. For example, YAP interacts with HIF1α and regulates the transcription of various genes involved in aerobic glycolysis such as *GLUT1, HK2, ALDOA, LDHA*, and *PKM2* (129, 130). The YAP-TEAD complex interacts with p65 and upregulates the transcription of *HK2* (131). YAP associates with c-Myc and regulates *PGAM1* mRNA expression (132). Besides, YAP directly regulates transcription of *GLUT3*, and PKM2 enhances *GLUT3* expression by interacting with YAP (126). In addition to direct transcriptional regulation, YAP also indirectly upregulates *HK2* and *PFKFB3* expression and glycolysis via the lncRNA *BCAR4*-GLI2 axis (133).

## The Role of YAP/TAZ in Lipid Metabolism

**Figure 4:**
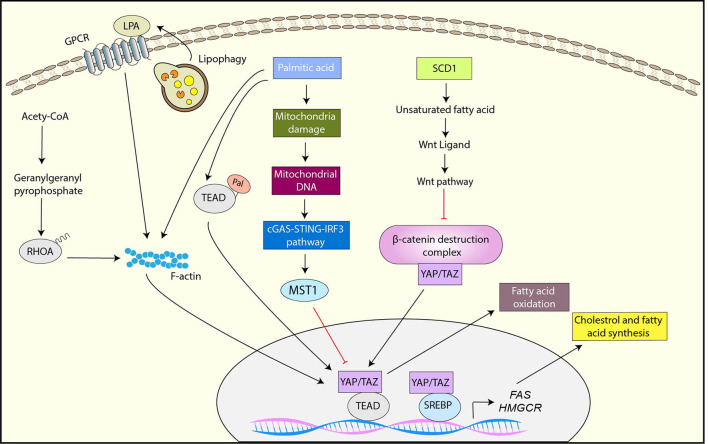
YAP/TAZ regulation by lipid metabolism and YAP/TAZ-targets for Lipid metabolism. SCD1-mediated unsaturated fatty acid activates YAP/TAZ by inhibiting the β-catenin destruction complex via Wnt activation. Palmitic acid inhibits YAP through the mitochondrial damage-mediated cGAS-STING-IRF3-MST1 pathway while it activates YAP-TAZ via palmitoylation of TEAD or F-actin rearrangement. The mevalonate pathway activates YAP/TAZ through geranylgeranylation of RHOA. Lipophagy is also associated with YAP activation likely through the upregulation of LPA, a ligand of GPCR. YAP interacts with SREBP1 and SREBP2 and enhances their transcriptional activity toward *FAS* and *HMGCR*. YAP also enhances fatty acid oxidation.

Another key metabolic reprograming that is critical for cancer progression is adaptation of lipid metabolism (134). Cancer cells require high levels of lipid to maintain their growth and proliferation, and exhibit elevated *de novo* synthesis or uptake of lipid compared to normal cells (134). A growing body of evidence has shown that YAP/TAZ is not only activated by the dysregulated lipid metabolism pathways but also plays a role in the adaptation of lipid metabolism in cancer (Figure 4)

*De novo* fatty acid synthesis is activated in cancer cells for the maintenance of membrane synthesis and generation of signaling molecules (135). Stearoyl-CoA-desaturase 1 (SCD1) is an enzyme that synthesizes monounsaturated fatty acids, and a study has shown that the inhibition of SCD1 downregulates YAP/TAZ expression, nuclear localization, and activity, suggesting that monounsaturated fatty acids are crucial for YAP function (136). The study has also indicated that SCD1 activates YAP/TAZ through the inhibition of the β-catenin destruction complex, which is likely induced by the upregulation of lipid modified Wnt proteins (136). Palmitic acid is a common saturated fatty acid in organisms. It has been reported that palmitic acid inhibits YAP by upregulating MST1, thereby inhibiting endothelial cell proliferation, migration, and angiogenesis (137). Mechanistically, palmitic acid induces mitochondrial damage, which results in releasing mitochondrial DNA (mtDNA) to the cytoplasm. The cytoplasmic mtDNA further activates cGAS–STING–IRF3 signaling, which subsequently induces *MST1* transcription (137). In contrast to endothelial cells, free fatty acids (FFA) including palmitic acid activate YAP via F-actin rearrangement in pancreatic β-cells (138). Notably, CTGF induced by YAP plays a role in protection of β-cells from FFA-induced apoptosis (138).

Protein S-palmitoylation is the post-translational modification by which palmitate is attached to cysteine residues of proteins, leading to protein association with membrane, localization changes, and/or functional alterations. Several studies have demonstrated that TEAD is palmitoylated and TEAD palmitylation is critical for YAP/TAZ function (139–141). Although TEAD palmitolaylation does not affect TEAD localization or membrane binding, it is important for the TEAD-YAP/TAZ interaction or proper TEAD folding and stability (139–141). Interestingly, TEAD is autopalmitoylated under physiological conditions (139). Moreover, a recent study has shown that TEAD palmitoylation is regulated by cell density (140). Mechanistically, high cell density induces NF2-dependent downregulation of fatty acid synthase (FASN) and acetyl-CoA carboxylase (ACC), which are the crucial enzymes for *de novo* biosynthesis of palmitate (140). Moreover, high cell density upregulates the expression of depalmitoylases including *APT2* and *ABHD17A*, which may contribute to TEAD depalmitoylation and subsequent TEAD downregulation (140). Therefore, these results suggest that TEAD palmitoylation serves as the potential regulatory mechanism of YAP/TAZ in response to cellular fatty acid metabolism.

The mevalonate pathway is another lipid metabolic pathway to synthesize sterols and isoprenoids from acetyl-CoA. The intermediate products of the mevalonate pathway including farnesyl pyrophosphate and geranylgeranyl pyrophosphate serve as substrates for protein farnesylation and geranylgeranylation, which plays a significant role in protein localization and function (142). Several studies have revealed that YAP/TAZ is regulated by the mevalonate pathway (96, 143, 144). These studies have further identified the underlying mechanism, in which the activation of YAP/TAZ by the mevalonate pathway is mediated by geranylgeranylation of RHOA and subsequent RHOA activation (96, 143, 144).

Lipophagy is the autophagic degradation process of lipid droplets, and a recent study has shown that oxidized low-density lipoprotein (oxLDL) lipophagy is associated with YAP activation and hepatocellular carcinoma (HCC) progression (145). In brief, endoplasmic reticulum-residential protein, Nogo-B is upregulated by the CD36-mediated oxLDL uptake, and Nogo-B interacts with ATG5 to promote oxLDL lipophagy, resulting in YAP activation likely through the upregulation of lysophosphatidic acid (LPA), which is a ligand of GPCR (145).

In addition to YAP/TAZ regulation by lipid metabolism, recent studies have shown that YAP/TAZ regulates lipid metabolism. The sterol regulatory element-binding proteins (SREBPs) are transcription factors that control the expression of enzymes involved in fatty acid and cholesterol biosynthesis (146). It has been reported that YAP interacts with the nuclear forms of SREBP1 and SREBP2 and enhances their transcriptional activities toward fatty acids synthase (FAS) and 30-hydroxylmethyl glutaryl coenzyme A reductase (HMGCR) (147). Also, the study has shown that the activation of LATS1 or inhibition of YAP reduced hepatic steatosis and hyperlipidaemia in diet-induced diabetic mice (147).

Fatty acids oxidation (FAO) is the metabolic pathway that degrade fatty acid to supply ATP and acetyl-CoA and the activity of FAO is promoted in various cancer types (148). Recently, it has been proved that knocking-down YAP reduces FAO while YAP overexpression activates it (149). In addition, the study has indicated that YAP-mediated metabolic shift to FAO is critical for lymph nodes metastasis in a melanoma mouse model (149).

## The Role of YAP/TAZ in Amino Acid Metabolism

**Figure 5:**
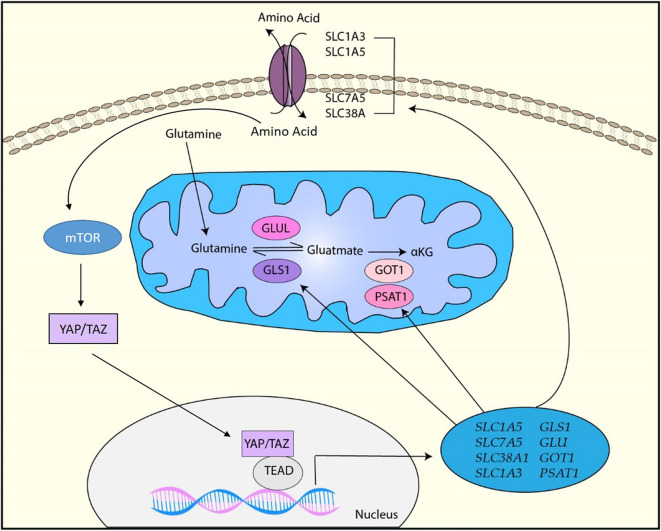
YAP/TAZ-targets for amino acid metabolism. YAP/TAZ regulates amino acid metabolism by upregulating various factors involved in amino acid transportation such as SLC1A3, SLC1A5, SCL7A5, and SLC38A1 as well as glutamine metabolism by upregulating GLS1, GLUL, GOT1, and PSAT1. Moreover, upregulation of amino acids induces the activation of mTOR, which also activates YAP/TAZ through various mechanisms.

In cancer, amino acid metabolism is also dysregulated. In particular, because glutamine is a key amino acid involved in energy generation in TCA cycle, biosynthesis of essential molecules such as amino acid, nucleotide, and fatty acid, control of redox homeostasis, and regulation of cell signaling (150), enhanced glutamine uptake and glutaminolysis are essential for cancer cell proliferation and survival (151). YAP/TAZ has been shown to be involved in glutamine metabolism by upregulating various genes involved in it (Figure 5).

It has been reported that YAP/TAZ directly regulates the mRNA expression of glutamine transporters *SLC38A1* and *SLC7A5* in HCC and these transporters are required for YAP/TAZ-mediated proliferation of HCC cells (152). In a zebra fish model, YAP has been shown to reprogram glutamine metabolism and enhance liver tumor formation (153). Briefly, YAP upregulates glutamine synthetase (GLUL) expression and activity, resulting in the upregulation of glutamine and nucleotide biosynthesis (153). In pulmonary vascular cells, YAP/TAZ upregulates the expression of glutaminase 1 (GLS1) that is the enzyme regulating the first step in the glutaminolysis by generating glutamate from glutamine (154). Physiologically, endothelial cell proliferation is enhanced by vascular stiffness in pulmonary hypertension. YAP/TAZ is activated by mechanical cues from stiff ECM and results in endothelial cell proliferation by upregulating GLS1-mediated glutaminolysis (154). It has been revealed that EphA2 induces YAP/TAZ activation through RHOA activation, and activated YAP/TAZ promotes glutaminolysis through upregulation of GLS1 and SLC1A5 in HER2-positive breast cancer cells (155). This study has also showed that *YAP/TAZ* expression positively correlates with *GLS1* and *SLC1A5* expression in human breast cancer patient samples (155). Another study with breast cancer cell lines has indicated that YAP/TAZ reprograms metabolism of breast cancer cells to glutamine-dependent one by inducing glutamic-oxaloacetic transaminase (GOT1) and phosphoserine aminotransferase (PSAT1), which are the key enzymes to convert glutamate to α-ketoglutarate (α-KG) (156). Thus, these studies demonstrated that the YAP/TAZ-mediated glutamine metabolism plays a role in breast cancer progression. Moreover, a recent study has demonstrated that mechanical cues from stiff ECM activates glutamine metabolism though YAP/TAZ activation in both cancer cells and cancer-associated fibroblasts (CAFs) (157). YAP/TAZ upregulates not only GLS1 to increase glutamine metabolism, but also SLC1A3 to exchange amino acids between cancer cells and CAFs (157). Cancer cells utilize CAFs-derived aspartate for the nucleotide biosynthesis pathway to maintain their proliferation while CAFs use cancer cell-derived glutamate for the glutathione pathway to maintain redox homeostasis (157). Thus, the study has indicated that YAP/TAZ regulates tumor growth by coordinating non-essential amino acids flux within the tumor niche.

In addition to glutamine metabolism, YAP/TAZ is also associated with the regulation of other amino acids. For example, YAP/TAZ increases leucine uptake by upregulating high-affinity leucine transporter LAT1 (158). Moreover, a study has suggested that YAP/TAZ activates serine metabolism (159). Upregulation of amino acids leads to the activation of the mammalian target of rapamycin (mTOR) signaling pathway, which is crucial for cell growth and survival (160). Numerous studies have shown that YAP/TAZ is closely associated with mTOR signaling through amino acids. For example, YAP/TAZ upregulates mTOR activity through the upregulation of amino acid transporters (152, 158). In addition, YAP activates mTOR by downregulating PTEN (161). Alternatively, the mTOR pathway has been shown to control YAP/TAZ activity through various mechanisms such as inhibition of autophagy, MST1, and AMOTL2, suggesting that cellular amino acids can regulate YAP/TAZ via activation of mTOR (162–164).

## Potential Link Between Metabolic Regulation and Metastasis By YAP and TAZ

Growing evidence has indicated that metabolic reprograming plays a critical role in metastasis (165). As mentioned in the earlier section, metastasis consists of multiple steps, in which cancer cells need to reprogram cellular metabolism to adapt to the drastic changes of environment. In addition, it has been revealed that metabolic reprogramming is associated with the phonotypic changes of cancer cells to gain specific ability required for metastasis (3).

Glycolysis is upregulated in metastatic cancer cells compared to non-metastatic ones (166), and the inhibition of glycolysis attenuates metastasis (167). EMT is a critical step for cancer cells to initiate metastasis and associated with cell migration and invasion ability, and glycolysis has been shown to be closely associated with EMT (168, 169). Induction of EMT in pancreatic ductal adenocarcinoma by tumor necrosis factor-α (TNF-α) and transforming growth factor-β (TGF-β) simultaneously reprograms glucose metabolism to enhance glycolysis (168). Moreover, EMT regulator Snail silences the expression of fructose-1,6-biphosphatase (FBP1), which is a rate-limiting enzyme in gluconeogenesis (170). Loss of FBP1 enhances glycolysis and reduces OXPHOS, while, FBP1 inhibits tumorigenesis *in vivo* (170). Because YAP/TAZ is activated by high levels of glucose and can regulate both EMT and glycolysis, it may coordinate glycolysis and EMT to initiate metastasis.

**Figure 6:**
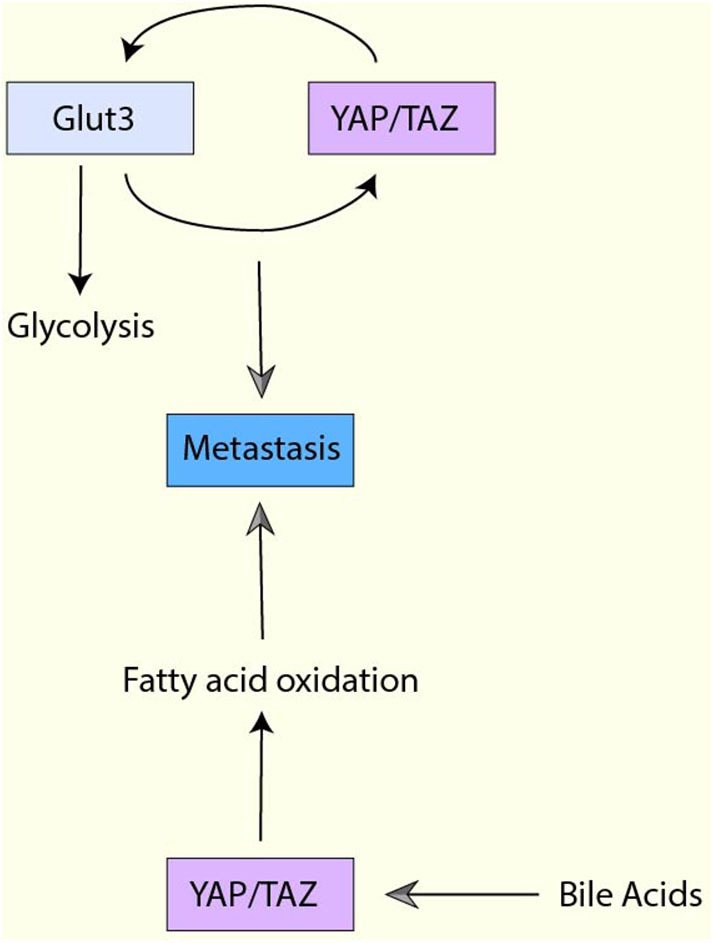
The interplay between YAP/TAZ-mediated metabolism and metastasis. Glut3 overexpression increases YAP/TAZ expression, and Glut3 promotes cell migration, invasion, and tumor sphere formation YAP/TAZ-dependently. Moreover, YAP/TAZ also induces Glut3 expression, and both YAP/TAZ and Glut3 are required for metastasis. YAP activation enhances fatty acid oxidation (FAO) and the enhanced FAO is required for lymph node metastasis. Moreover, YAP in the cancer cells is activated by bile acids, which is accumulated to high levels in the metastatic lymph nodes.

Indeed, one report have demonstrated that YAP/TAZ-mediated glucose metabolism is vital for metastasis. In brief, glycolysis and glucose transporter Glut3 are upregulated in metastatic colorectal cancer cells, and Glut3 expression is correlated with poor survival in colorectal cancer patients (126). Interestingly, Glut3 overexpression increases YAP/TAZ expression while Glut3-induced cell migration, invasion, and tumor sphere formation activity are abrogated by YAP knockdown (126). Moreover, YAP induces Glut3 expression in colorectal cancer cells, and both YAP and Glut3 are required for high-fat/sucrose diet-mediated metastasis in a xenograft mouse model (126). Thus, YAP/TAZ and Glut3 regulates each other to control glycolysis and metastasis in colorectal cancer (Figure 6). In addition to glycolysis, OXHOS and activated mitochondria have been shown to play a role in metastasis (171, 172). Moreover, a study has revealed that specific metabolic adaptations are required for breast cancer cells to metastasize to particular organs (173). Liver-metastatic cancer cells show enhanced glycolysis and reduced OXPHOS while bone- or brain-metastatic cancer cells display enhanced OXPHOS (173). So far, it is uncertain whether YAP/TAZ is involved in organ-specific metastasis. However, considering YAP/TAZ promotes glycolysis, it may contribute to organ-specific metastasis.

Several reports have indicated that the lipid metabolic pathways are also associated with metastasis. For example, in ovarian cancer, fatty acid incorporation via CD36 is required for cell migration and invasion *in vitro* and metastasis *in vivo* (174). In contrast, in A549 non-small lung cancer cells, lipogenesis plays a negative role in TGF-β-induced EMT and metastasis (175). Therefore, the role of lipid metabolism may be context-dependent. In mevalonate-mediated YAP activation, YAP regulates RHAMM, which upregulates breast cancer invasion and migration activity (96). Moreover, the recent study using a melanoma mouse model has also showed that YAP-mediated FAO is critical for metastasis (149) (Figure 6). In this model, YAP activation enhances FAO and the enhanced FAO is required for lymph node metastasis. Additionally, YAP in the cancer cells is activated by bile acids, which is accumulated to high levels in the metastatic lymph nodes (149).

Glutamine metabolism has also been reported to be engaged in metastasis. For example, glutaminolytic enzyme, glutamate dehydrogenase 1 (GDH1), has been shown to contribute to anoikis resistance and promote metastasis (176). Mechanistically, GDH1 enhances α-KG, which further regulates energy balance through the CamKK2-AMPK pathway after cell detachment (176). In colorectal cancer tissues, GLS1 expression is positively correlated with lymph node metastasis and advanced clinical stage (177). Moreover, GLS1 is induced by hypoxia via HIF1α in colorectal cancer cell lines, and knockdown of GLS1 reduces metastasis in a xenograft mouse model with colorectal cancer cells (177). So far, there is no direct evidence that YAP/TAZ-mediated gluaminolysis contributes to metastasis. However, because YAP/TAZ can regulate GLS1 expression and glutaminolysis, it is likely that YAP/TAZ-mediated glutaminolysis contributes to metastasis. Moreover, although the GDH1-CamKK2-AMPK pathway confers resistance to anoikis, YAP/TAZ is known to be negatively regulated by AMPK. Thus, the glutamine metabolism-mediated anti-apoptotic function may be context-dependent. Obviously further studies are necessary to address the role of YAP/TAZ in glutaminolysis-mediated metastasis.

## Concluding Remarks

Recent advances in targeted therapy and immunotherapy have significantly improved survival rates of cancer patients. However, majority of metastatic cancer is still challenging to be cured. Therefore, it is important to understand the pathways that are involved in metastasis and identify the potential druggable targets. As we described here, YAP/TAZ is activated by high nutrient conditions, and regulates multiple aspects of metastasis as well as cancer metabolism. Thus, YAP/TAZ would be a promising drug target for metastatic cancer. Although no effective clinical drugs targeting YAP/TAZ are available, multiple direct and indirect inhibitors for YAP/TAZ have been developed (178). Verteporfin, which is a photosensitizer for photodynamic therapy, has been known to inhibit YAP/TAZ activity by blocking YAP/TAZ-TEAD interaction and downregulating YAP/TAZ expression (179, 180). Indeed, multiple studies have demonstrated that verteporfin inhibits YAP/TAZ-dependent expression of the genes related to glycolysis and glutaminolysis and subsequent glycolysis and glutaminoslysis *in vitro* and *in vivo* (126, 129, 153, 154, 157, 181). In addition to verteporfin, a peptide mimicking VGLL4 has also been shown to inhibit YAP-TEAD interaction and suppress tumor growth *in vitro* and *in vivo* (42). Moreover, several specific small molecule inhibitors for YAP-TEAD interaction have been developed (182–184). These inhibitors may have better therapeutic effects with less toxicity than verteporfin because of their specificity. As an alternative approach, YAP/TAZ upstream regulators, in particular protein kinases and other enzymes, would serve as potential drug targets for attenuating YAP/TAZ activity (178). However, YAP/TAZ is regulated by many upstream regulators and the regulatory mechanism may be dependent on cell context. Therefore, it is critical to identify key YAP/TAZ regulators in each tumor for targeting it. Moreover, the expression of YAP/TAZ-target genes would serve as a biomarker to monitor efficacy of therapy.

In recent years, numerous studies have demonstrated the role of YAP/TAZ in metabolism and metastasis. However, there are several critical questions that need to be answered to understand YAP/TAZ-mediated metabolism and metastasis.

Because YAP/TAZ is a multifunctional transcriptional regulator, it is expected to be involved in various steps of metastasis. However, it is still uncertain exactly how YAP/TAZ is regulated during metastasis. For example, EMT is considered as a first step for metastasis, but mesenchymal to epithelial transition (MET) also plays a role in it (185). Moreover, cancer cells reprogram their metabolism between high glycolysis and high OXPHOS during metastasis (3). Therefore, YAP/TAZ activity needs to be controlled strictly during metastasis. Cancer cells are exposed to diverse types of mechanical cues, and changes of nutrient and oxygen during metastasis, which may contribute to the regulation of YAP/TAZ and coordinate the cancer progression and metastasis. Indeed, some mechanical cues from ECM have been shown to promote EMT and metastasis as well as alter cellular metabolism (154, 157, 186, 187). In addition to LATS-mediated phosphorylation, YAP/TAZ is regulated by the interaction with other transcription factors and other proteins. Thus, during the metastasis and metabolic alterations, YAP/TAZ may change its binding partners so that it alters its function according to the need of microenvironment. In addition, various extracellular metabolites may affect YAP/TAZ binding partners and signaling, and contribute to metabolic adaptation and metastasis. Moreover, several reports have suggested that YAP also exhibits context-dependent tumor suppressor function (188, 189). The complicated metabolic regulation by YAP/TAZ may be associated with its tumor suppressor function. Thus, the understanding of the diverse role of YAP/TAZ in different cellular contexts may lead to a better therapeutic strategy to target metastatic cancer.

## Author Contributions

HY wrote the manuscript. GT made the figures and contributed to editing the manuscript. All authors contributed to the article and approved the submitted version.

## Conflict of Interest

The authors declare that the research was conducted in the absence of any commercial or financial relationships that could be construed as a potential conflict of interest.
